# Effectiveness of a Mindfulness-Based Mobile Intervention for Improving Perinatal Mental Health and Reducing Depression During Pregnancy: Randomized Controlled Trial

**DOI:** 10.2196/75630

**Published:** 2025-10-02

**Authors:** Minjung Kim, Jin Young Park, Sehwan Park, Kyungmi Chung, Hee Young Cho, Gangho Do, Kyungun Jhung

**Affiliations:** 1 Institute of Behavioral Science in Medicine, Yonsei University College of Medicine, Yonsei University Health System, Seoul, Republic of Korea; 2 Department of Psychiatry, Yongin Severance Hospital, Yonsei University College of Medicine, Yonsei University Health System, Yongin, Republic of Korea; 3 Center for Digital Health, Yongin Severance Hospital, Yonsei University College of Medicine, Yonsei University Health System, Yongin, Republic of Korea; 4 Medical Research Team, Digital Medic Co, Seoul, Republic of Korea; 5 Department of Obstetrics and Gynecology, Seoul National University College of Medicine, Seoul, Republic of Korea; 6 Department of Psychiatry, Catholic Kwandong University International St. Mary’s Hospital, Catholic Kwandong University College of Medicine, 25 Simgok-ro 100 beongil, Incheon, 22711, Republic of Korea, +82 1090563807

**Keywords:** perinatal period, depression, mindfulness, mobile app intervention, mindfulness-based intervention

## Abstract

**Background:**

Pregnancy is a vulnerable period for women, with an increased risk of mental health issues such as depression and anxiety. The perinatal period is particularly important, as maternal mental health significantly impacts maternal physical health, pregnancy outcomes, postnatal outcomes, and fetal and infant development. Psychotherapeutic interventions for depression are essential, especially given the limitations of medication use during pregnancy. However, perinatal women experiencing depression often encounter barriers to accessing these interventions. Digital health interventions may offer a promising approach to overcome these barriers.

**Objective:**

This study aimed to (1) evaluate the effectiveness of *Avecmom*, a self-help, mindfulness-based mobile intervention, in reducing depression severity among pregnant women with mild to moderate depression; (2) examine its impact on emotional well-being and maternal-fetal attachment; and (3) explore whether the effect on mental well-being is mediated by increased mindfulness and reduced depression severity.

**Methods:**

Participants were recruited both online and offline. Of 158 screened individuals, 90 met the inclusion criteria and were randomly assigned to either the intervention or control group. The intervention group used both the *Avecmom* and *Big 4+* apps, while the control group used only the *Big 4+* app. Depressive symptoms, anxiety, and stress were assessed as primary outcomes at baseline and postintervention. Mindfulness, mental well-being, positive and negative emotions, and maternal-fetal attachment were assessed as secondary outcomes. Analysis of covariance (ANCOVA) was conducted with postintervention scores as the dependent variable, adjusting for baseline scores. To estimate the mechanism of change following *Avecmom* mobile app use, the bootstrapping technique with PROCESS Macro Model 6 was employed, applying the difference score from postintervention to baseline.

**Results:**

A total of 86 pregnant women (intervention group: n=42; control group: n=42) completed the study and were included in the final analysis, reflecting an overall dropout rate of 4% (4/90). At postintervention, the intervention group demonstrated lower depression severity scores (*F*_1,84_=5.69; *P*=.02; partial *ƞ*^2^=0.06) and higher scores of mindfulness (*F*_1,84_=11.18; *P*=.001; partial *ƞ*^2^=0.12), maternal-fetal attachment (*F*_1,84_=5.54; *P*=.02; partial *ƞ*^2^=0.06), mental well-being (*F*_1,84_=8.79; *P*=.004; partial *ƞ*^2^=0.10), and positive affect (*F*_1,84_=7.21; *P*=.009; partial *ƞ*^2^=0.08) compared to the control group. Serial mediation analysis revealed that *Avecmom* app use influenced mental well-being through sequential improvements in mindfulness and decreases in depression severity (standardized *β*=.33; Boot SE=0.12, 95% CI 0.12-0.58).

**Conclusions:**

Mindfulness-based mobile app interventions tailored for pregnant women may be more effective than self-monitoring alone in improving perinatal depression. These findings suggest that mindfulness-based mobile app interventions may help improve mental well-being in pregnant women, potentially by enhancing mindfulness and reducing depression severity.

## Introduction

The prevalence of mental health problems is higher among pregnant women than in the general population [1]. Perinatal women are particularly vulnerable to depression, with an estimated global occurrence rate of 15%‐65% [2,3]. Perinatal depression is associated with adverse maternal outcomes, including poor nutrition and physical health, disrupted sleep, negative cognition, suicidal ideation, substance use, and reduced social support [4,5]. The effects of depression can persist beyond the perinatal period, increasing the risk of depression and negatively impacting maternal physical and mental health, as well as fetal development and postnatal outcomes such as premature birth and impaired infant development [6,7].

Mindfulness practice is increasingly being recognized as an effective approach for alleviating perinatal mental health problems. Mindfulness is defined as “paying attention in a particular way: on purpose, in the present moment, and nonjudgmentally” [8]. Mindfulness meditation enhances mental health by increasing awareness and altering responses to negative emotions. Psychological interventions incorporating mindfulness practices have demonstrated effectiveness in reducing stress and negative emotions [9-12]. Research indicates that mindfulness-based interventions (MBIs) can also enhance positive psychological outcomes, including subjective well-being and emotional regulation ability [13]. Furthermore, MBIs have been shown to be effective in treating a range of psychiatric disorders, including mood disorders, such as depression and anxiety, as well as insomnia and addiction [14].

Studies on MBIs in perinatal women have demonstrated positive effects on perinatal mental health [15]. Psychotherapeutic approaches are particularly crucial in treating depression during pregnancy, as many women are hesitant to receive pharmacotherapy due to concerns about medication safety for the fetus. However, several barriers impede access to psychotherapeutic interventions for perinatal women [16]. Women experiencing depression during pregnancy often face social stigma and apprehension about being labeled as “bad mothers” [17]. A survey of 401 pregnant women aged 20-40 years revealed that, while 17.2% reported experiencing depression, only 8.7% received interventions [18]. Even when women seek appropriate treatment, they often continue to face barriers such as limited access to specialized psychotherapeutic resources, time constraints, and financial difficulties.

Digital health presents a promising approach to overcoming these barriers through the use of technologies such as online platforms and mobile apps. Digital health is increasingly being used in the treatment of various mental health disorders, including mood disorders, substance abuse, and trauma-related disorders [19]. It offers particular benefits to pregnant women who may be reluctant to seek in-person care, alleviating concerns about stigma and discrimination while overcoming limitations of time and space [20].

Research on the use of digital health in maternal mental health has steadily increased. Digital mental health interventions have shown promise for addressing perinatal depression and anxiety [21,22]. Green et al [23] demonstrated significant improvements in sleep problems, anxiety, and stress among perinatal women who used a commercial mindfulness app. However, many existing studies, including those mentioned, were not specifically designed to address the unique needs of perinatal women. Furthermore, only a limited number of studies have examined mobile MBI apps specifically tailored for this population [24,25]. Among these studies, interventions demonstrated not only reductions in depression and anxiety but also improvements in autonomy and acceptance compared to control conditions.

These interventions often require either a lengthy duration or multiple sessions, such as typical 8-week programs or 20 sessions spread over 4 weeks. Such extended durations or high session counts can contribute to low completion rates, sometimes falling below 52.4% [24,25]. Given that participant attrition from psychotherapeutic interventions can compromise their effectiveness [26,27], ensuring participant retention is crucial in their design. To address this challenge, our research team developed *Avecmom*, a self-help mobile MBI app that provides 10‐15 minutes of mindfulness training within a 4-week program, designed for ease of completion [28]. A prior assessment of *Avecmom*’s impact on prenatal anxiety, depression symptoms, and stress among pregnant women indicated a decrease in anxiety levels within the experimental group, while the waitlist control group (CG) showed no significant changes; however, the overall dropout rate was approximately 30%.

While the effectiveness of mobile MBI apps has been assessed, these evaluations have often been limited to general populations, thereby highlighting the need for further research in clinical settings for the treatment of perinatal depression. Therefore, this study aimed to investigate the effectiveness of a 4-week mindfulness-based self-help app in reducing depression severity and improving mental well-being among perinatal women with mild to moderate depression. To ensure a more rigorous evaluation of the *Avecmom* app, we selected a self-monitoring app as the active control condition, in contrast to the waitlist CG used in the previous study [28]. Self-monitoring is widely recognized for its capacity to enhance self-awareness [29], which can support improved self-guided care for mental health issues and contribute to the reduction of negative emotions [30,31].

Building on these insights, this study aimed to evaluate the effectiveness of the *Avecmom* app in reducing depression severity, anxiety, and stress among perinatal women experiencing mild to moderate depression, compared to a self-monitoring control. Secondary outcomes, including mindfulness, positive and negative affect, maternal-fetal attachment, and mental well-being, were also assessed to provide a comprehensive understanding of the app’s impact. This study also investigated the potential mechanisms by which the *Avecmom* app might alleviate depression among perinatal women.

## Methods

### Trial Design and Procedure

This study was a single-blinded randomized controlled trial (RCT) with a pre-post assessment design (trial registration: KCT0008887; Clinical Research Information Service [CRIS]). Participants were recruited via online and poster advertisements, and from the outpatient department of Catholic Kwandong University International St. Mary’s Hospital, from October 2023 to December 2023. Eligible participants provided informed consent and underwent a screening process, leading to the exclusion of those who did not meet the inclusion criteria. To maintain single-blind conditions, participants were blinded to their assigned study condition (intervention group [IG] or CG).

Participants were randomly assigned to either the IG or CG prior to completing the initial online assessment. Randomization was performed using a list of random numbers generated via 4-block randomization in R (version 4.0.2). This list, securely stored as an encrypted file to ensure allocation concealment, was managed by a researcher independent of participants’ recruitment and assessment, who assigned participants to either the IG or CG based on their registration order. After the assignment, participants completed an online survey collecting sociodemographic, medical, pregnancy-related, and psychiatric information, as well as baseline measures for primary and secondary outcomes.

Upon completion of the baseline assessment, both the IG and CG commenced their respective 4-week interventions. At the conclusion of this period, participants were notified of study completion and requested to complete an online survey for postintervention assessment. The postintervention assessment was designed to mirror the baseline assessment.

### Sample Size

The sample size was determined using G*Power software, version 3.1.9.4 (Franz Faul, University of Kiel, Germany). Based on power analysis aiming to detect differences between 2 groups over time, the calculation assumed a large effect size and an α level of .05, with a statistical power of 95%. This yielded a required sample size of 70 participants (35 per group). To account for potential attrition, which has been reported to reach up to 25% in a previous study [28], the recruitment target was set at 88 participants at baseline.

### Participants

Potential participants were screened according to the following inclusion criteria: (1) pregnant women aged 19‐65 years; (2) experiencing mild to moderate depression, as indicated by a score of 10‐20 on the depression subscale of the Depression, Anxiety, and Stress Scale-21 (DASS-21) [32,33] or a score of 9‐11 on the Edinburgh Postnatal Depression Scale (EPDS) [34,35]; (3) possession of a smartphone compatible with the required mobile app; (4) meeting the minimum operating system requirements for the mobile app; and (5) ability to actively use the mobile app and provide feedback regarding its functionality.

The exclusion criteria were as follows: (1) gestational age exceeding 34 weeks at the time of enrollment or expected delivery within 6 weeks of enrollment; (2) diagnosis of severe psychiatric disorders, including bipolar disorder, major depression disorder with psychotic features, posttraumatic stress disorder, and obsessive-compulsive disorder; (3) presence of other severe medical or psychiatric conditions that would impede study participation or safety; and (4) inability to download a mobile app.

### Interventions

To investigate whether a mobile MBI app is more effective than self-monitoring alone, the IG used both the *Avecmom* app and the *Big 4+* app for 4 weeks, whereas the CG used only the *Big 4+* app for the same duration.

The *Avecmom* app (Digital Medic Co, Ltd), developed by Cho et al [36], is a freely accessible self-help mobile MBI app used exclusively in the IG. This intervention is designed to support the self-management of depressive symptoms among pregnant women. The *Avecmom* app aims to enhance mindful awareness and compassion to help women manage perinatal stress, depression, and anxiety. It comprises 4 modules involving mindfulness training, structured for completion within 4 weeks. The 4 core mindfulness trainings—breathing meditation, body scan, emotion-awareness meditation, and loving-kindness meditation—unlocked successively. Each subsequent module became accessible after participants completed the primary training within the preceding module two or more times. Participants were instructed to practice mindfulness at least twice a week using the *Avecmom* app. To support adherence, they were encouraged to set personalized in-app meditation reminders, and daily automated notifications were sent on days when nonengagement was detected to promote continued engagement.

The mindfulness training content offered by the *Avecmom* app was specifically designed to alleviate discomfort among perinatal women. It introduces basic mindfulness training focusing on breathing and bodily sensations in modules 1‐2 and then progresses to mindfulness training integrated with techniques of emotional and cognitive behavioral therapy in modules 3‐4. To address pregnancy-specific discomforts, such as concerns about fear of miscarriage or increased physical strain [37], the training also included content aimed at fostering maternal-fetal attachment and managing pregnancy-related bodily sensations. This content was preliminarily reviewed by perinatal women to ensure it was appropriately tailored to their needs. The detailed content and usage process of the *Avecmom* app are described in the report by Park et al [28], and the app is freely available to the public on both iOS and Android platforms.

The *Big 4+* app (Digital Medic Co, Ltd) is a self-monitoring app that includes single-item assessments and a diary function. It enables users to monitor various psychological states, such as mood, sleep duration, appetite, and other relevant psychological parameters, providing users with easy and personalized access to their self-reported data. Users of the *Big 4+* app were encouraged to record their status once daily using the single-item measures provided within the app. Upon completing the measurement, users received self-feedback through data visualizations presented in graphs. The *Big 4+* app is accessible on both iOS and Android platforms, and all participants in both the IG and CG were asked to use the *Big 4+* app.

For intervention delivery, the IG participants were provided with URLs for downloading both the *Avecmom* app and *Big 4+* app, along with manuals on how to install and use them. Participants’ understanding of app usage, initially guided by the manuals, was verified by a responsible researcher via phone or in-app messenger. Throughout the intervention period for the IG, a separate researcher continuously monitored participants’ engagement rates via the *Avecmom* app’s administrator page. In-app notifications were sent to encourage participants who did not meet the predefined engagement standard (eg, practicing at least twice a week). Similarly, the CG received a URL to download the *Big 4+* app with its respective manuals, and their understanding of the app’s use was verified by a researcher via phone or in-app messenger.

### Measures

#### Sociodemographic and App Use Data

Sociodemographic data were collected from both the IG and CG, encompassing information such as education level, planned pregnancy status, smoking and alcohol use, history of depression, and other relevant medical conditions. Additionally, data on the duration of participants’ engagement with audio-guided mindfulness training within the *Avecmom* app were collected from the IG to examine the relationship between clinical variables and mindfulness training duration.

#### Primary Outcomes

##### EPDS Score

The EPDS [34,35] assesses various signs of clinical depression, including guilt, sleep disturbance, lack of energy, anhedonia, and suicidal ideation in postnatal women. Although originally developed for detecting depression in postnatal women, it has also become the most widely used tool to detect depression in pregnant women [38]. The EPDS consists of 10 items rated on a 4-point scale ranging from 0 to 3, with 7 items being reverse-scored. A total EPDS score of 9 is considered an appropriate cutoff value for identifying potential depression. A previous study reported good internal reliability (Cronbach α=0.84) [35]. Our study also found satisfactory internal reliability (Cronbach *α*=0.79).

##### Center for Epidemiologic Studies Depression Scale-Revised Score

The Center for Epidemiologic Studies Depression Scale-Revised (CESD-R) [39,40] was designed to measure depression by adapting the original CES-D scale [41] to align with the diagnostic criteria of the Diagnostic and Statistical Manual of Mental Disorders, Fourth Edition. It comprises 20 items measured on a 5-point scale (ranging from 0 to 4), with total possible scores ranging from 0 to 80. The cutoff score is 16. In a standardization study conducted in Korea, the reliability of the CESD-R was excellent (Cronbach *α*=0.98) [40]. In our study, Cronbach α was 0.91.

##### DASS-21 Score

The DASS-21 [32,33] is a shortened form of the original DASS-42 [42], consisting of 21 items rated on a 4-point scale ranging from 0 to 3. The DASS-21 includes 3 subscales: depression, anxiety, and stress. A higher sum of the scores indicates greater symptom severity. A previous study involving the DASS-21 reported excellent reliability of the total scale (Cronbach *α*=0.94), with good reliability for the subscales (depression: Cronbach *α*=0.88; anxiety: Cronbach *α*=0.80; stress: Cronbach *α*=0.87) [33]. In our study, the reliability of the total scale was also excellent (Cronbach *α*=0.93), with good reliability for the subscales (depression: Cronbach *α*=0.87; anxiety: Cronbach *α*=0.81; stress: Cronbach *α*=0.85).

### Secondary Outcomes

#### Cognitive and Affective Mindfulness Scale-Revised Score

The Cognitive and Affective Mindfulness Scale-Revised (CAMS-R), developed by Feldman et al [43,44], is a self-assessment scale used to measure mindfulness. It uses a 4-point scale ranging from 1 to 4, with 1 reverse-scored item. This scale includes 3 mindfulness subscales: awareness, attention, and acceptance. Higher scores indicate a greater level of mindfulness. The reliability reported in a previous study was acceptable for the total scale (Cronbach *α*=0.73) [44], and our study found that the reliability of the total scale was excellent (Cronbach *α*=0.90).

#### Positive Affect and Negative Affect Schedule Score

The Positive Affect and Negative Affect Schedule (PANAS) [45,46] has 20 items, each rated on a 5-point scale (ranging from 1 to 5). It originally included 10 items measuring positive affect and 10 items measuring negative affect. During the translation and validation into Korean, 1 item (“alert”) was loaded under negative affect, resulting in a discrepancy in the number of items for each affect compared with the original one. Negative affect items were reverse scored. Higher scores indicate greater levels of positive or negative affect. In our study, the internal consistency reliabilities were good for negative affect and positive affect (negative affect: Cronbach *α*=0.90; positive affect: Cronbach *α*=0.82).

#### Maternal-Fetal Attachment Scale Score

The Maternal-Fetal Attachment Scale (MFAS) [47,48] consists of 23 questions rated on a 4-point scale ranging from 1 to 4. Maternal-fetal attachment refers to the degree of interaction and emotional connection between the mother and the fetus. Total scores range from 23 to 92, with higher scores indicating greater maternal-fetal attachment. In terms of reliability, a previous study reported a good reliability coefficient (Cronbach *α*=0.88) [48], and our study reported an excellent reliability coefficient (Cronbach *α*=0.91).

#### Mental Health Continuum Short Form

The Mental Health Continuum Short Form (MHC-SF) [49,50] is a scale designed to measure the level of mental well-being. It consists of 14 questions rated on a 6-point scale ranging from 0 (never) to 5 (daily). This scale includes 3 constructs of mental well-being: emotional, social, and psychological. Higher scores indicate a higher degree of mental well-being. The internal reliability in a previous study was found to be excellent (Cronbach *α*=0.93) [50], and in our study, it was also excellent (Cronbach *α*=0.92).

### Statistical Analysis

All statistical analyses were conducted using IBM SPSS Statistics for Windows, version 25 (IBM Corp), with the significance level set at an α level of .05 (2-tailed). Initially, independent *t* tests and chi-square tests were performed to compare baseline sociodemographic and clinical variables between the IG and CG. Analysis of covariance (ANCOVA) was then employed to analyze the pre-post data for primary and secondary outcomes. In these analyses, postintervention scores served as the dependent variable, with corresponding baseline scores included as covariates. This approach was chosen because it provides unbiased intervention effect estimates and enhances estimation precision [51]. Specifically, ANCOVA was conducted to examine group differences in scores of the EPDS, CESD-R, DASS-21, CAMS-R, PANAS, MFAS, and MHC-SF.

To further explore relationships and underlying mechanisms of change, change scores for all clinical variables were calculated by subtracting baseline scores from postintervention scores. We then examined correlations among these change scores across all participants. Additionally, correlations were investigated between the clinical change scores and the recorded duration of audio-guided mindfulness training within the *Avecmom* app, specifically for participants in the IG. Subsequently, serial mediation analysis was performed using PROCESS MACRO by Hayes, Model 6 (version 4.2) [52]. This analysis examined the serial mediating effects of changes in CAMS-R and CESD-R scores on the relationship between *Avecmom* app use and changes in MHC-SF scores. The mediation model was tested using 95% CIs generated from 5000 bootstrap samples.

### Ethical Considerations

This study was conducted in accordance with the principles of the Declaration of Helsinki. Prior to commencing the study, all participants provided informed consent. All study protocols were approved by the Institutional Review Board of Catholic Kwandong University International St. Mary’s Hospital (IRB number: IS23EIMI0054). To ensure participant privacy and data security, all collected data were deidentified prior to analysis and stored in password-encrypted files, accessible only to authorized research staff. Participants received compensation of KRW 100,000 (US $71.35) to cover transportation expenses.

## Results

### Recruitment and Enrollment

**Figure 1.**
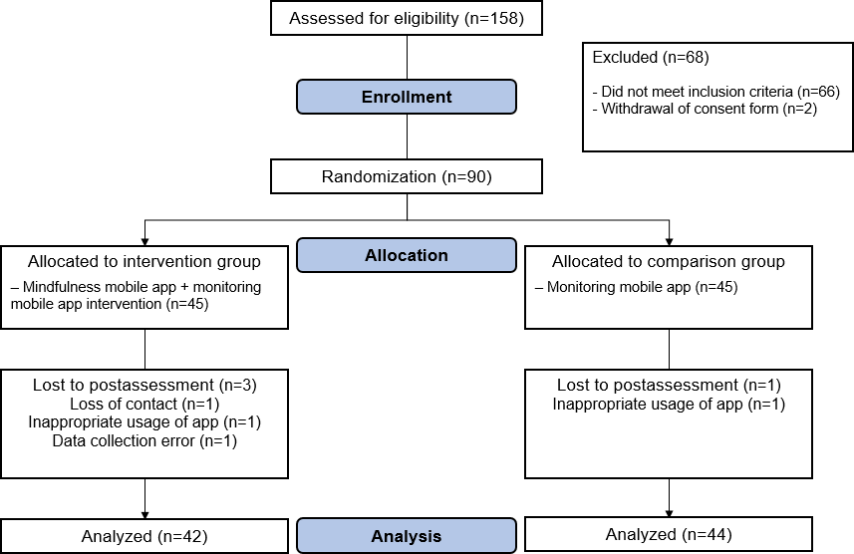
Participant enrollment flow chart.

As depicted in Figure 1, a total of 158 participants were screened for eligibility. Of these, 68 were excluded based on the established criteria, and 90 were subsequently randomized to either the IG or CG. During the postintervention assessment period, 4 participants were excluded from the study (3 from the IG and 1 from the CG) due to reasons such as loss of contact, insufficient adherence to the intervention protocol (defined as a *Big 4+* app response rate below 70%), and data collection errors. Finally, 86 participants completed the study and were included in the final analysis. The CONSORT-EHEALTH checklist is provided in Checklist 1.

### Group Differences in Sociodemographic Variables

Participants in the IG (n=42) had a mean age of 34.10 (SD 3.04) years, while those in the CG (n=44) had a mean age of 33.80 (SD 3.42) years. There was no significant difference in age between the 2 groups (*t*_84_=0.43; *P*=.67). Similarly, at baseline, the mean gestational period was 25.43 (SD 6.77) weeks for the IG and 23.40 (SD 6.88) weeks for the CG, with no significant difference observed (*t*_84_=1.37; *P*=.17). Table 1 summarizes the group differences in other sociodemographic variables. Consistent with the age and gestational period findings, no significant differences were observed across any of these variables, including education level, pregnancy planning, smoking habits, alcohol use, history of depression, and other relevant medical problems.

**Table 1.** Comparison of sociodemographic variables between the control and intervention groups.

Characteristic	Control group (n=44)	Intervention group (n=42)	*t* test (*df*)	Chi-square (*df*)	*P* value
Age (years), mean (SD)	33.80 (3.42)	34.10 (3.04)	0.43 (84)	—^a^	.67
Gestational period (weeks), mean (SD)	23.40 (6.88)	25.43 (6.77)	−1.37 (84)	—	.17
Education, n (%)			—	2.0 (2)	.36
High school or lower	2 (4)	3 (7)			
College	37 (84)	30 (71)			
Graduated school	5 (11)	9 (21)			
Planned pregnancy, n (%)			—	0.2 (1)	.68
Yes	39 (89)	36 (86)			
No	5 (11)	6 (14)			
Smoking, n (%)			—	0.5 (1)	.50
Yes	6 (14)	8 (19)			
No	38 (86)	34 (81)			
Alcohol use, n (%)			—	1.5 (1)	.23
Yes	6 (14)	10 (24)			
No	38 (86)	32 (76)			
Depression history, n (%)			—	1.2 (1)	.27
Yes	9 (20)	13 (31)			
No	35 (80)	29 (69)			
Medical problems, n (%)			—	1.3 (1)	.25
Yes	8 (18)	12 (29)			
No	36 (82)	30 (71)			

a Not applicable.

### Homogeneity Test of Clinical Variables

The homogeneity test revealed no significant differences in the clinical variables between the IG and CG at baseline. Descriptive statistics and *t* values are presented in Table 2.

**Table 2.** Results of the homogeneity test for clinical variables.

Scale and group				Value, mean (SD)		Skewness	Kurtosis	*t* test (*df*)	*P* value
EPDS^a^								−0.70 (84)	.48
Control group (n=44)				12.73 (5.30)		0.50	−0.47		
Intervention group (n=42)				12.05 (3.55)		−0.27	−0.03		
CESD-R^b^								0.62 (84)	.54
Control group (n=44)				18.86 (13.38)		0.97	0.82		
Intervention group (n=42)				18.98 (10.89)		0.72	0.39		
DASS-21^c^									
Total score								−0.02 (84)	.99
Control group (n=44)				16.32 (12.05)		1.07	0.42		
Intervention group (n=42)				16.29 (6.81)		0.76	0.92		
Anxiety								−0.29 (84)	.77
Control group (n=44)				4.07 (3.93)		1.06	0.22		
Intervention group (n=42)				3.86 (2.55)		0.69	0.23		
Depression								−0.76 (84)	.45
Control group (n=44)				5.43 (4.50)		1.18	0.83		
Intervention group (n=42)				4.83 (2.56)		0.47	0.12		
Stress								0.90 (84)	.37
Control group (n=44)				6.81 (4.45)		0.70	−0.26		
Intervention group (n=42)				7.60 (3.51)		0.83	0.58		
CAMS-R^d^								0.43 (84)	.67
Control group (n=44)				23.06 (4.72)		0.25	−0.31		
Intervention group (n=42)				23.52 (5.03)		−0.18	−1.34		
MHC-SF^e^								0.37 (84)	.71
Control group (n=44)				26.98 (11.95)		0.84	0.54		
Intervention group (n=42)				27.90 (10.98)		0.21	−0.96		
MFAS^f^								0.62 (84)	.54
Control group (n=44)				58.61 (12.82)		−0.19	−0.17		
Intervention group (n=42)				60.12 (9.40)		−0.06	0.73		
PANAS^g^									
Positive affect								0.94 (84)	.35
Control group (n=44)				19.66 (5.27)		0.55	−0.45		
Intervention group (n=42)				20.83 (6.26)		−0.06	−1.07		
Negative affect								0.82 (84)	.42
Control group (n=44)				25.07 (9.15)		0.73	−0.55		
Intervention group (n=42)				26.60 (8.16)		0.43	0.22		

a EPDS: Edinburgh Postnatal Depression Scale.

b CESD-R: Center for Epidemiologic Studies Depression Scale-Revised.

c DASS-21: Depression Anxiety Stress Scale-21.

d CAMS-R: Cognitive and Affective Mindfulness Scale-Revised.

e MHC-SF: Mental Health Continuum Short Form.

f MFAS: Maternal-Fetal Attachment Scale.

g PANAS: Positive Affect and Negative Affect Schedule.

### Group Comparisons for Clinical Variables

**Figure 2.**
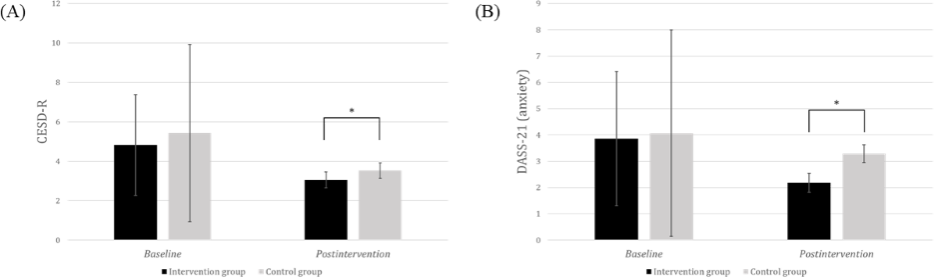
Group differences in baseline mean scores and adjusted postintervention mean scores for the primary outcomes of (A) CESD-R and (B) DASS-21 (anxiety) between the intervention group and control group. CESD-R: Center for Epidemiologic Studies Depression Scale-Revised; DASS-21: Depression Anxiety Stress Scale-21. *P<.05.

An ANCOVA was conducted to compare postintervention scores between the groups, adjusting for baseline scores. The detailed ANCOVA results are presented in Table 3, and the significant findings for each variable in the IG and CG are visually represented in Figures2  3, respectively.

**Figure 3.**
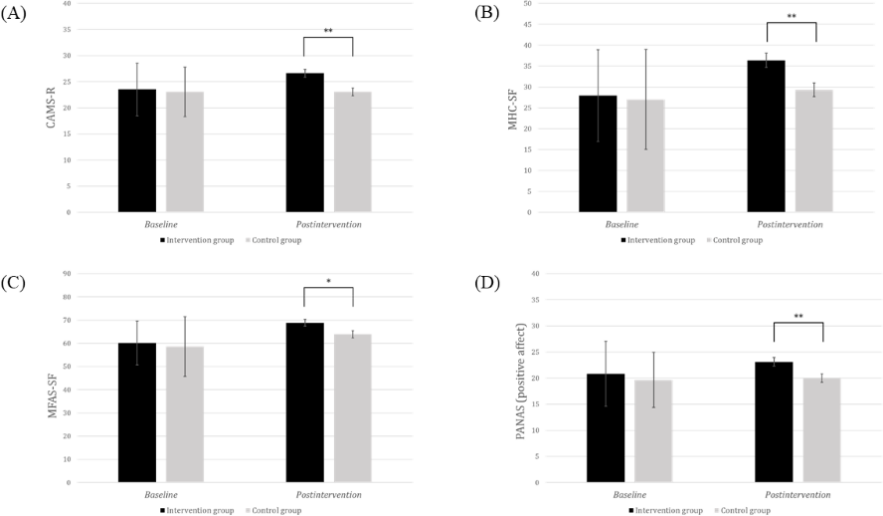
Group differences in baseline mean scores and adjusted postintervention mean scores for the secondary outcomes of (A) CAMS-R, (B) MHC-SF, (C) MFAS, and (D) PANAS (positive affect) between the intervention group and control group. CAMS-R: Cognitive and Affective Mindfulness Scale-Revised; MFAS: Maternal-Fetal Attachment Scale; MHC-SF: Mental Health Continuum Short Form; PANAS: Positive Affect and Negative Affect Schedule. *P<.05, **P<.01.

**Table 3.** Group differences in posttest scores after adjusting for baseline scores.

Scale and group			Posttest score, mean (SD)	Adjusted posttest score, adjusted mean (SE)	*F* test (*df*)	*P* value	Partial *ƞ*^2^
EPDS^a^					0.99 (1, 84)	.32	0.01
Control group (n=44)			8.93 (5.41)	8.77 (0.62)			
Intervention group (n=42)			7.71 (3.66)	7.89 (0.63)			
CESD-R^b^					5.69 (1, 84)	.02	0.06
Control group (n=44)			17.20 (13.46)	17.24 (1.28)			
Intervention group (n=42)			12.90 (8.27)	12.87 (1.31)			
DASS-21^c^							
Total score					1.84 (1, 84)	.18	0.02
Control group (n=44)			12.11 (9.41)	12.11 (1.06)			
Intervention group (n=42)			10.05 (6.74)	10.06 (1.08)			
Anxiety					5.28 (1, 84)	.02	0.06
Control group (n=44)			3.32 (2.91)	3.29 (0.34)			
Intervention group (n=42)			2.14 (1.86)	2.18 (0.35)			
Depression					0.74 (1, 84)	.39	0.01
Control group (n=44)			3.68 (3.57)	3.53 (0.39)			
Intervention group (n=42)			2.90 (2.64)	3.06 (0.40)			
Stress					0.55 (1, 84)	.46	0.01
Control group (n=44)			5.11 (3.83)	5.29 (0.45)			
Intervention group (n=42)			5.00 (3.20)	4.81 (0.46)			
CAMS-R^d^					11.18 (1, 84)	.001	0.12
Control group (n=44)			22.93 (5.01)	23.07 (0.74)			
Intervention group (n=42)			26.78 (6.49)	26.64 (0.76)			
MHC-SF^e^					8.79 (1, 84)	.004	0.10
Control group (n=44)			29.02 (12.52)	29.32 (1.66)			
Intervention group (n=42)			37.67 (14.02)	36.36 (1.70)			
MFAS^f^					5.54 (1, 84)	.02	0.06
Control group (n=44)			63.41 (11.14)	63.92 (1.48)			
Intervention group (n=42)			69.43 (11.69)	68.90 (1.51)			
PANAS^g^							
Positive affect					7.21 (1, 84)	.009	0.08
Control group (n=44)			19.68 (6.25)	20.00 (0.81)			
Intervention group (n=42)			23.45 (6.17)	23.12 (0.83)			
Negative affect					0.05 (1, 84)	.82	0.00
Control group (n=44)			22.20 (8.59)	22.58 (0.98)			
Intervention group (n=42)			23.31 (6.98)	22.91 (1.01)			

a EPDS: Edinburgh Postnatal Depression Scale.

b CESD-R: Center for Epidemiologic Studies Depression Scale-Revised.

c DASS-21: Depression Anxiety Stress Scale-21.

d CAMS-R: Cognitive and Affective Mindfulness Scale-Revised.

e MHC-SF: Mental Health Continuum Short Form.

f MFAS: Maternal-Fetal Attachment Scale.

g PANAS: Positive Affect and Negative Affect Schedule.

For primary outcomes, the IG demonstrated significantly lower depression severity (CESD-R; *F*_1,84_=5.69; *P*=.02; partial *ƞ*^2^=0.06) and anxiety (DASS-21 anxiety subscale; *F*_1,84_=5.28; *P*=.02; partial *ƞ*^2^=0.06) compared to the CG. Conversely, no significant differences were observed between the groups for depressive symptoms (EPDS and DASS-21 depression subscale scores), stress (DASS-21 stress subscale scores), or total DASS-21 scores.

Regarding secondary outcomes, the IG exhibited significantly higher levels of mindfulness (CAMS-R; *F*_1,84_=11.18; *P*=.001; partial *ƞ*^2^=0.12), mental well-being (MHC-SF; *F*_1,84_=8.79; *P*=.004; partial *ƞ*^2^=0.10), and maternal-fetal attachment (MFAS; *F*_1,84_=5.54; *P*=.02; partial *ƞ*^2^=0.06) compared to the CG. Positive affect scores were significantly higher in the IG than in the CG (PANAS-PA; *F*_1,84_=7.21; *P*=.009; partial *ƞ*^2^=0.08), while negative affect (PANAS-NA) scores did not show significant differences between the groups.

### Usage Patterns of the *Avecmom* App and Their Clinical Correlates

Participants in the IG were instructed to engage with the *Avecmom* app at least twice weekly to progress to subsequent sessions. The app was used, on average, 12.60 times (SD 2.19), with individual usage ranging from 10 to 23 times. The mean total duration of app use was approximately 110.57 (SD 18.08) minutes. Module-specific mean usage durations (in minutes) were as follows: module 1, 23.66 (SD 5.66); module 2, 33.65 (SD 5.49); module 3, 27.93 (SD 4.48); module 4, 25.32 (SD 6.65). The temporal distribution of app usage indicated that most sessions occurred in the morning (6:00 AM to 12:00 PM; 231/552 sessions, 41.9%), followed by the afternoon (12:00 PM to 6:00 PM; 157/552 sessions, 28.4%), evening (6:00 PM to 12:00 AM; 130/552 sessions, 23.6%), and late night (12:00 AM to 6:00 AM; 34/552 sessions, 6.2%).

Correlation analyses were conducted between the duration of engagement with each *Avecmom* app module and the clinical variables, with details presented in Table 4. No significant correlations were found between clinical variables and the durations of modules 1, 2, and 3. Significant correlations were observed between the duration of module 4 (which primarily features loving-kindness meditation) and negative affect (*r*=−0.39; *P*=.01) and mindfulness (*r*=0.33; *P*=.04). Additionally, a positive correlation trend was noted between the duration of module 4 and both mental well-being (*r*=0.30; *P*=.05) and maternal-fetal attachment (*r*=0.30; *P*=.05), though these correlations did not reach statistical significance.

**Table 4.** Correlation between the duration of each module of the *Avecmom* app and change in the scores of clinical variables in the intervention group**.**

Variable		Duration of module 1	Duration of module 2	Duration of module 3	Duration of module 4	Total duration
EPDS^a^						
*r*		0.06	0.27	0.21	−0.19	0.08
*P* value		.73	.09	.19	.22	.62
CESD-R^b^						
*r*		0.05	−0.07	0.12	−0.12	−0.02
*P* value		.76	.66	.44	.44	.90
DASS-21^c^						
*r*		0.12	0.04	0.09	−0.11	0.03
*P* value		.44	.82	.56	.47	.85
CAMS-R^d^						
*r*		0.09	0.04	0.01	0.33	0.16
*P* value		.58	.80	.97	.04	.31
MHC-SF^e^						
*r*		−0.07	−0.02	−0.04	0.30	0.08
*P* value		.68	.90	.82	.05	.64
MFAS^f^						
*r*		0.11	0.10	0.07	0.30	0.19
*P* value		.49	.52	.68	.05	.22
PANAS^g^						
Positive affect						
*r*		−0.08	0.05	−0.08	0.02	−0.02
*P* value		.64	.78	.60	.89	.89
Negative affect						
*r*		−0.30	−0.01	0.05	−0.39	−0.23
*P* value		.06	.94	.77	.01	.15

a EPDS: Edinburgh Postnatal Depression Scale.

b CESD-R: Center for Epidemiologic Studies Depression Scale-Revised.

c DASS-21: Depression Anxiety Stress Scale-21.

d CAMS-R: Cognitive and Affective Mindfulness Scale-Revised.

e MHC-SF: Mental Health Continuum Short Form.

f MFAS: Maternal-Fetal Attachment Scale.

g PANAS: Positive Affect and Negative Affect Schedule.

### Mediating Effect of Mindfulness on the Relationship Between *Avecmom* App Use and Mental Well-Being

**Figure 4.**
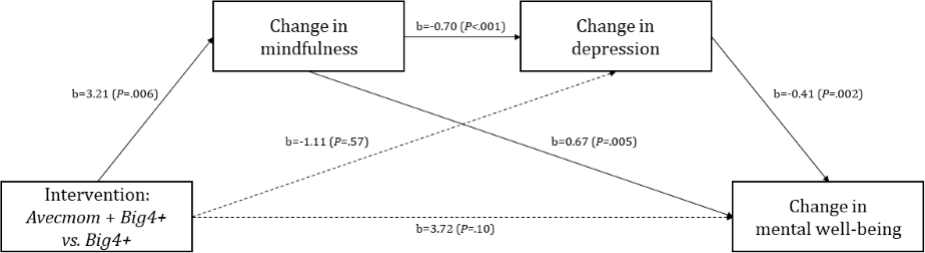
Serial mediation model of change for mental well-being in perinatal women with depression. Total effect: b=7.25; SE 2.51, 95% CI 2.26-12.25; Direct effect: b=3.72; SE 2.27, 95% CI −0.79 to 8.24.

Serial mediation analysis was conducted using PROCESS Macro by Hayes, Model 6 (version 4.2), to investigate whether changes in mindfulness and depression severity serially mediated the relationship between the intervention (the combined use of the *Avecmom* app and *Big 4+* app) and changes in mental well-being. As depicted in Figure 4, the mediation model revealed several significant paths. The intervention led to significantly greater changes in mindfulness compared to the control condition (b=3.48; *P*=.002). Increased mindfulness was significantly associated with a reduction in depression severity (b=−0.82; *P*<.001). This enhanced mindfulness also directly influenced changes in mental well-being, showing a positive effect (b=0.78; *P*=.003). Changes in depression severity were negatively associated with changes in mental well-being (b=−0.28; *P*=.03).

Table 5 presents the specific indirect effects. The results revealed a significant serial indirect effect of the intervention on the changes in mental well-being through sequential changes in mindfulness and depression severity (CESD-R; standardized *β*=.07; Boot SE=0.05, 95% CI 0.003-0.19). Additionally, a significant indirect effect was found for the path from the intervention through changes in mindfulness alone to changes in mental well-being (standardized *β*=.23; Boot SE=0.11, 95% CI 0.06-0.49). In summary, *Avecmom* app use was found to improve mental well-being in perinatal women. This improvement appears to be primarily driven by the enhancement of mindfulness, which, in turn, contributes to a reduction in depression and directly fosters mental well-being.

**Table 5.** Indirect effect of the intervention on the improvement of mental well-being through changes in mindfulness and depression.

Indirect effect	Standardized *β*	Boot SE	95% CI	
			Boot LLCI^a^	Boot ULCI^b^
Total indirect effect	0.33	0.12	0.12	0.58
Intervention→CAMS-R^c^→MHC-SF^d^	0.23	0.11	0.06	0.49
Intervention→CESD-R^e^→MHC-SF	0.04	0.05	−0.07	0.14
Intervention→CAMS-R→CESD-R→MHC-SF	0.07	0.05	0.003	0.29

a LLCI: lower limit confidence interval.

b ULCI: upper limit confidence interval.

c CAMS-R: Cognitive and Affective Mindfulness Scale-Revised.

d MHC-SF: Mental Health Continuum Short Form.

e CESD-R: Center for Epidemiologic Studies Depression Scale-Revised.

## Discussion

### Principal Results

This study evaluated the effectiveness of the *Avecmom* app, a mobile MBI app specifically designed for perinatal women, in reducing depression severity among pregnant women with mild to moderate depression. The findings indicated that the use of the *Avecmom* app significantly reduced depression severity and anxiety levels compared to self-monitoring alone. Furthermore, the use of the mobile MBI app enhanced mindfulness, overall mental well-being, maternal-fetal attachment, and positive affect compared to self-monitoring alone. Notably, module 4, which focused on loving-kindness meditation, showed potential for greater improvements in mindfulness, negative affect, maternal-fetal attachment, and overall mental health compared to other modules, such as breathing meditation, body scan, and emotion-awareness meditation. Serial mediation analysis showed that *Avecmom* app use enhanced mindfulness, which, in turn, reduced depression severity and ultimately improved overall mental well-being.

The current findings demonstrate the effectiveness of the *Avecmom* app in reducing depression severity and anxiety among perinatal women with mild to moderate depression. Despite its relatively brief protocol, with weekly engagement averaging less than 30 minutes, the mobile MBI app produced meaningful changes in both depression severity and anxiety, comparable to other MBIs with more extended protocols [53,54]. These results are particularly noteworthy given the rigorous RCT design and the inherent benefits of self-monitoring. Self-monitoring, a common active component in various digital mental health interventions, is known to enhance self-awareness, thereby potentially contributing to improved outcomes. Our study distinctively showed that the combined approach of self-monitoring and mindfulness training yielded significantly greater benefits than self-monitoring alone.

According to the Monitor and Acceptance Theory proposed by Lindsay and Creswell [55], mindfulness inherently involves both monitoring and acceptance components. In the context of our study, the CG effectively practiced monitoring alone through the *Big 4+* app, while the IG engaged in both monitoring and acceptance through the combined use of the *Avecmom* and *Big 4+* apps. This distinction likely contributed to the observed differences between the groups. Supporting this, a previous study found that engaging in both monitoring and acceptance improved positive affect more substantially than practicing either component in isolation [56]. Consistent with this, our current findings also revealed a more positive affect in the IG compared to the CG, further suggesting the effectiveness of mindfulness as an intervention strategy for enhancing positive affect.

An improvement in maternal-fetal attachment was also observed in this study. Maternal-fetal attachment, defined as the emotional bond between a mother and her fetus [57], has been consistently reported to be negatively associated with perinatal depression symptoms and maternal anxiety [58,59]. Furthermore, strong maternal-fetal attachment is known to positively influence the social and emotional development of infants after birth [60]. These findings suggest that the *Avecmom* app may exert a long-term beneficial effect by influencing not only users’ depression severity but also other critically related developmental factors. Therefore, future studies should be designed to evaluate the long-term effectiveness of the *Avecmom* app and to determine whether additional booster sessions are necessary to sustain its beneficial effects.

Among the various sessions of mindfulness training offered within the *Avecmom* app, correlation analysis revealed that engagement with module 4 (loving-kindness meditation) had a positive correlational trend with maternal-fetal attachment. Loving-kindness meditation aims to cultivate unconditional love and kindness toward oneself and others [61], which may have contributed to enhancing maternal-fetal attachment in this population. While previous studies have reported an association between loving-kindness meditation and enhanced mental health outcomes, such as positive emotions, its specific effectiveness in perinatal depression has not been extensively explored [62,63]. Furthermore, loving-kindness meditation is not yet frequently featured in mobile MBI apps designed for maternity care. The current findings thus suggest that integrating loving-kindness meditation into perinatal care may be particularly beneficial for mothers experiencing depression and, by extension, for their children.

Another notable finding of this study was that the overall total time spent on mindfulness training was not significantly associated with changes in clinical variables. This finding aligns with a meta-review suggesting that increased hours of mindfulness training might not necessarily yield proportionally better outcomes [64]. The recommended weekly training duration within the *Avecmom* app is relatively short, at approximately 30 minutes. Nonetheless, most participants exhibited improvements in psychiatric outcomes, even when adhering to the minimum standard of training twice a week. However, it is premature to definitely conclude that there is no relationship between the extent of mindfulness training and the improvement in psychological outcomes, as some studies have reported that a longer mindfulness training duration may be associated with greater benefits [65,66]. In particular, long-term meditators have been shown to have different brain connectivity and amygdala activation levels in response to emotional stimuli compared to those engaging in mindfulness training for the first time [67,68]. Therefore, further research is needed to examine the effects of increasing the frequency and duration of practice.

This study further explored how the *Avecmom* app could enhance the overall mental well-being of pregnant women with depression, focusing on the sequential and direct roles of changes in mindfulness and depression severity. The serial mediation analysis demonstrated that *Avecmom* app use contributed to improved overall mental well-being. Crucially, our findings from this model illuminated how enhanced mindfulness, a primary outcome of the intervention, appears to impact mental well-being not through a single, isolated path, but rather through a sophisticated interplay of effects. Specifically, increased mindfulness was observed to directly foster improvements in mental well-being. Simultaneously, it also sequentially contributed to a reduction in depression severity, which, in turn, further mediated the positive change in mental well-being. This finding aligns with broader evidence suggesting that mobile MBI apps may positively influence not only overall mental well-being but also a range of clinical variables that may impact it in diverse populations [69-71]. Consistent with these findings, previous discussions on the mechanisms of traditional MBIs have suggested that mindfulness training enhances mental well-being by increasing mindfulness and reducing reactivity to emotional stimuli [13]. By integrating these insights with our results, it appears that mobile MBI apps operate through mechanisms similar to those of traditional face-to-face MBIs.

Lastly, the dropout rate in this study was 4%, which is markedly lower than the 30% dropout rate reported in a previous study that used the same *Avecmom* app and a similar design [28]. One possible explanation for this discrepancy is the difference in participant characteristics; while the earlier study targeted the general perinatal population, this study specifically recruited participants experiencing mild to moderate depression. Compared to other mobile mindfulness interventions, the low dropout rate in this study may also be attributed to the relatively short intervention duration and the app’s high perceived user experience. The *Avecmom* app features 4 mindfulness modules designed to be completed within a 4-week period, with each module unlocking after participants complete its primary training session two or more times. Participants were instructed to complete at least two training sessions per week. Compared to other mobile MBIs, this structured yet flexible design likely reduced participant burden and improved engagement, which consequently led to a positive influence on user satisfaction ratings. In a prior development study of the *Avecmom* app, participants rated its usability using the USE Questionnaire [28]. On a 7-point Likert scale, the average scores were as follows: usefulness, 5.4 (SD 0.79); satisfaction, 5.6 (SD 0.79); ease of learning, 6.4 (SD 0.67); and ease of use, 5.9 (SD 0.68), consistently indicating above-average evaluations across all domains.

In summary, this study addressed a significant research gap by evaluating the effectiveness of a 4-week mobile MBI app tailored for pregnant women experiencing mild to moderate depression, a population often overlooked in digital health research. Unlike many previous studies that predominantly relied on commercial apps, such as Headspace and Calm, or focused on general and nonclinical populations [23,24,72-75], the *Avecmom* app was uniquely designed for pregnant women with depression and demonstrated significant effectiveness, including a reduction in depression severity and anxiety symptoms despite the short duration. Notably, a previous review indicated limited effects of digital interventions on anxiety [76]. The *Avecmom* app showed promise as an effective and accessible option for perinatal women, particularly given their limited medication options. Its digital nature also enables automated data sharing with health care professionals and integration with electronic health records, which can support coordinated care and continuous monitoring, thereby suggesting its potential utility within stepped care models for perinatal depression. To achieve broader clinical implementation, however, future research would importantly need to evaluate its effectiveness when used concurrently with pharmacotherapy and further explore its potential to serve as either a first-line self-help intervention or an adjunct to established treatments, ultimately facilitating its systematic integration into health care infrastructure.

### Limitations

A primary limitation of this study is the absence of an untreated CG, which precludes definitively distinguishing between changes attributable to the intervention and those occurring naturally over time, especially given the dynamic psychological landscape during pregnancy [77]. Thus, there is a need for future research incorporating designs that enable such crucial distinctions. Another limitation lies in the integrated nature of the active components, namely self-monitoring and loving-kindness meditation. However, both are recognized for their independent benefits [78-82]. Furthermore, as an initial investigation of a newly developed intervention, the applicability of the findings of this study in broader clinical settings is limited by the lack of evidence regarding the long-term effects and the effectiveness in individuals with severe depression. Therefore, future research is crucial to address these limitations for the *Avecmom* app to achieve broader clinical implementation.

### Conclusions

The findings of this study demonstrate the effectiveness of a mobile MBI app in enhancing the mental health of pregnant women with mild to moderate depression, compared to self-monitoring alone. Specifically, the intervention led to enhanced mindfulness that was found to influence mental well-being through multiple pathways, including directly fostering mental well-being and sequentially contributing to a reduction in depression severity, which then improved mental well-being. Furthermore, its relatively short protocol and high accessibility contributed to higher adherence. Mobile MBI apps offer a promising avenue to overcome barriers to accessing traditional psychotherapeutic treatments, presenting an effective and accessible option for perinatal women experiencing depression.

## Supplementary material

10.2196/75630 Checklist 1 CONSORT-EHEALTH checklist (V 1.6.1).

## Abbreviations

ANCOVA: analysis of covariance
CAMS-R: Cognitive and Affective Mindfulness Scale-Revised
CESD-R: Center for Epidemiologic Studies Depression Scale-Revised
CG: control group
DASS-21: Depression, Anxiety, and Stress Scale-21
EPDS: Edinburgh Postnatal Depression Scale
IG: intervention group
MBI: mindfulness-based intervention
MFAS: Maternal-Fetal Attachment Scale
MHC-SF: Mental Health Continuum Short Form
PANAS: Positive Affect and Negative Affect Schedule
RCT: randomized controlled trial

## References

[1] Chauhan A, Potdar J (2022). Maternal mental health during pregnancy: a critical review. Cureus.

[2] O’Hara MW, Wisner KL (2014). Perinatal mental illness: definition, description and aetiology. Best Pract Res Clin Obstet Gynaecol.

[3] Dadi AF, Miller ER, Bisetegn TA, Mwanri L (2020). Global burden of antenatal depression and its association with adverse birth outcomes: an umbrella review. BMC Public Health.

[4] Dagher RK, Bruckheim HE, Colpe LJ, Edwards E, White DB (2021). Perinatal depression: challenges and opportunities. J Womens Health (Larchmt).

[5] Muzik M, Borovska S (2010). Perinatal depression: implications for child mental health. Ment Health Fam Med.

[6] Jahan N, Went TR, Sultan W (2021). Untreated depression during pregnancy and its effect on pregnancy outcomes: a systematic review. Cureus.

[7] Coussons-Read ME (2013). Effects of prenatal stress on pregnancy and human development: mechanisms and pathways. Obstet Med.

[8] Kabat-Zinn J (1994). Wherever You Go, There You Are: Mindfulness Meditation in Everyday Life.

[9] Kabat-Zinn J (1982). An outpatient program in behavioral medicine for chronic pain patients based on the practice of mindfulness meditation: theoretical considerations and preliminary results. Gen Hosp Psychiatry.

[10] Guendelman S, Medeiros S, Rampes H (2017). Mindfulness and emotion regulation: insights from neurobiological, psychological, and clinical studies. Front Psychol.

[11] de Vibe M, Bjørndal A, Fattah S, Dyrdal GM, Halland E, Tanner‐Smith EE (2017). Mindfulness‐based stress reduction (MBSR) for improving health, quality of life and social functioning in adults: a systematic review and meta‐analysis. Campbell Systematic Reviews.

[12] Teasdale JD, Segal ZV, Williams JM, Ridgeway VA, Soulsby JM, Lau MA (2000). Prevention of relapse/recurrence in major depression by mindfulness-based cognitive therapy. J Consult Clin Psychol.

[13] Keng SL, Smoski MJ, Robins CJ (2011). Effects of mindfulness on psychological health: a review of empirical studies. Clin Psychol Rev.

[14] Zhang D, Lee EKP, Mak ECW, Ho CY, Wong SYS (2021). Mindfulness-based interventions: an overall review. Br Med Bull.

[15] Leng LL, Yin XC, Ng SM (2023). Mindfulness-based intervention for clinical and subthreshold perinatal depression and anxiety: a systematic review and meta-analysis of randomized controlled trial. Compr Psychiatry.

[16] Webb R, Uddin N, Constantinou G (2023). Meta-review of the barriers and facilitators to women accessing perinatal mental healthcare. BMJ Open.

[17] Thornicroft G, Mehta N, Clement S (2016). Evidence for effective interventions to reduce mental-health-related stigma and discrimination. The Lancet.

[18] (2018). From pregnancy experience to consideration, culture, and support policy [Article in Korean]. Korea Population Health and Welfare Association.

[19] Philippe TJ, Sikder N, Jackson A (2022). Digital health interventions for delivery of mental health care: systematic and comprehensive meta-review. JMIR Ment Health.

[20] Koh J, Tng GYQ, Hartanto A (2022). Potential and pitfalls of mobile mental health apps in traditional treatment: an umbrella review. J Pers Med.

[21] Chen C, Wang X, Xu H, Li Y (2023). Effectiveness of digital psychological interventions in reducing perinatal depression: a systematic review of meta-analyses. Arch Womens Ment Health.

[22] Lewkowitz AK, Whelan AR, Ayala NK (2024). The effect of digital health interventions on postpartum depression or anxiety: a systematic review and meta-analysis of randomized controlled trials. Am J Obstet Gynecol.

[23] Green J, Neher T, Puzia M, Laird B, Huberty J (2022). Pregnant women’s use of a consumer-based meditation mobile app: a descriptive study. Digit Health.

[24] Carissoli C, Gasparri D, Riva G, Villani D (2022). Mobile well-being in pregnancy: suggestions from a quasi-experimental controlled study. Behav Inf Technol.

[25] Sun Y, Li Y, Wang J, Chen Q, Bazzano AN, Cao F (2021). Effectiveness of smartphone-based mindfulness training on maternal perinatal depression: randomized controlled trial. J Med Internet Res.

[26] Schindler A, Hiller W, Witthöft M (2013). What predicts outcome, response, and drop-out in CBT of depressive adults? a naturalistic study. Behav Cogn Psychother.

[27] Lebeau RT, Davies CD, Culver NC, Craske MG (2013). Homework compliance counts in cognitive-behavioral therapy. Cogn Behav Ther.

[28] Park S, Cho HY, Park JY, Chung K, Jhung K (2025). Development and evaluation of a mindfulness-based mobile intervention for perinatal mental health: randomized controlled trial. J Med Internet Res.

[29] Schueller SM, Neary M, Lai J, Epstein DA (2021). Understanding people’s use of and perspectives on mood-tracking apps: interview study. JMIR Ment Health.

[30] Caldeira C, Chen Y, Chan L, Pham V, Chen Y, Zheng K (2017). Mobile apps for mood tracking: an analysis of features and user reviews. AMIA Annu Symp Proc.

[31] Dubad M, Elahi F, Marwaha S (2021). The clinical impacts of mobile mood-monitoring in young people with mental health problems: the MeMO study. Front Psychiatry.

[32] Henry JD, Crawford JR (2005). The short-form version of the Depression Anxiety Stress Scales (DASS-21): construct validity and normative data in a large non-clinical sample. Br J Clin Psychol.

[33] Cha ES, Kim KH, Erlen JA (2007). Translation of scales in cross-cultural research: issues and techniques. J Adv Nurs.

[34] Cox JL, Holden JM, Sagovsky R (1987). Detection of postnatal depression. Development of the 10-item Edinburgh Postnatal Depression Scale. Br J Psychiatry.

[35] Kim YK, Hur JW, Kim KH, Oh KS, Shin YC (2008). Clinical application of Korean version of Edinburgh Postnatal Depression Scale. J Korean Neuropsychiatr Assoc.

[36] Cho HY (2021). Development of health promotion service for women in perinatal period using obstetrics chatbot and mental health app [Article in Korean]. Science On.

[37] Rallis S, Skouteris H, McCabe M, Milgrom J (2014). A prospective examination of depression, anxiety and stress throughout pregnancy. Women Birth.

[38] O’Connor E, Senger CA, Henninger ML, Coppola E, Gaynes BN (2019). Interventions to prevent perinatal depression: evidence report and systematic review for the US Preventive Services Task Force. JAMA.

[39] Eaton WW, Smith C, Ybarra M, Muntaner C, Tien A, Maruish ME (2004). The Use of Psychological Testing for Treatment Planning and Outcomes Assessment: Instruments for Adults.

[40] Lee S, Oh ST, Ryu SY (2016). Validation of the Korean version of Center for Epidemiologic Studies Depression Scale-Revised (K-CESD-R). Korean Journal of Psychosomatic Medicine.

[41] Radloff LS (1977). The CES-D scale: a self-report depression scale for research in the general population. Appl Psychol Meas.

[42] Lovibond SH, Lovibond PF (1995). Manual for the Depression Anxiety Stress Scales.

[43] Feldman G, Hayes A, Kumar S, Greeson J, Laurenceau JP (2007). Mindfulness and emotion regulation: the development and initial validation of the Cognitive and Affective Mindfulness Scale-Revised (CAMS-R). J Psychopathol Behav Assess.

[44] Cho Y (2009). The reliability and validity of a Korean version of the Cognitive and Affective Mindfulness Scale-Revised. Korean Journal of Clinical Psychology.

[45] Watson D, Clark LA, Tellegen A (1988). Development and validation of brief measures of positive and negative affect: the PANAS scales. J Pers Soc Psychol.

[46] Lee HH, Kim EJ, Lee MK (2003). A validation study of Korea Positive and Negative Affect Schedule: the PANAS scales. Korean Journal of Clinical Psychology.

[47] Cranley MS (1981). Development of a tool for the measurement of maternal attachment during pregnancy. Nurs Res.

[48] Park JH (2001). The effect of visual and verbal information by antenatal ultrasound on maternal-fetal attachment and self-care [Article in Korean]. https://www-riss-kr-ssl.access.yonsei.ac.kr/link?id=T8028328.

[49] Keyes CLM, Wissing M, Potgieter JP, Temane M, Kruger A, van Rooy S (2008). Evaluation of the mental health continuum-short form (MHC-SF) in setswana-speaking South Africans. Clin Psychol Psychother.

[50] Lim YJ, Ko Y, Shin H, Cho Y (2012). Psychometric evaluation of the Mental Health Continuum-Short Form (MHC-SF) in South Koreans [Article in Korean]. Korean Journal of Psychology.

[51] O’Connell NS, Dai L, Jiang Y (2017). Methods for analysis of pre-post data in clinical research: a comparison of five common methods. J Biom Biostat.

[52] Hayes AF (2013). Introduction to Mediation, Moderation, and Conditional Process Analysis: A Regression-Based Approach.

[53] Linardon J, Messer M, Goldberg SB, Fuller-Tyszkiewicz M (2024). The efficacy of mindfulness apps on symptoms of depression and anxiety: an updated meta-analysis of randomized controlled trials. Clin Psychol Rev.

[54] Wu J, Ma Y, Zuo Y (2021). Effects of mindfulness exercise guided by a smartphone app on negative emotions and stress in non-clinical populations: a systematic review and meta-analysis. Front Public Health.

[55] Lindsay EK, Creswell JD (2017). Mechanisms of mindfulness training: Monitor and Acceptance Theory (MAT). Clin Psychol Rev.

[56] Lindsay EK, Chin B, Greco CM (2018). How mindfulness training promotes positive emotions: dismantling acceptance skills training in two randomized controlled trials. J Pers Soc Psychol.

[57] Rubin BB, Matos M de, Trettim JP (2023). Which social, gestational and mental health aspects are associated to maternal-fetal attachment?. Rev Bras Saude Mater Infant.

[58] Rollè L, Giordano M, Santoniccolo F, Trombetta T (2020). Prenatal attachment and perinatal depression: a systematic review. Int J Environ Res Public Health.

[59] Anjarwati A, Koni Suryaningsih E (2021). The relationship between pregnancy-related anxiety and maternal-fetal attachment among primigravida. Open Access Maced J Med Sci.

[60] Borges Rubin B, Puchalski Trettim J, Coelho Scholl C (2022). Maternal-fetal attachment and social-emotional development in infants at 3 months of age: a population-based study in southern Brazil. Interpers Int J Pers Relatsh.

[61] Hofmann SG, Grossman P, Hinton DE (2011). Loving-kindness and compassion meditation: potential for psychological interventions. Clin Psychol Rev.

[62] Zeng X, Chiu CPK, Wang R, Oei TPS, Leung FYK (2015). The effect of loving-kindness meditation on positive emotions: a meta-analytic review. Front Psychol.

[63] Petrovic J, Mettler J, Cho S, Heath NL (2024). The effects of loving-kindness interventions on positive and negative mental health outcomes: a systematic review and meta-analysis. Clin Psychol Rev.

[64] Strohmaier S (2020). The relationship between doses of mindfulness-based programs and depression, anxiety, stress, and mindfulness: a dose-response meta-regression of randomized controlled trials. Mindfulness (N Y).

[65] Bowles NI, Davies JN, Van Dam NT (2022). Dose-response relationship of reported lifetime meditation practice with mental health and wellbeing: a cross-sectional study. Mindfulness (N Y).

[66] Liu W, Yuan J, Wu Y (2024). A randomized controlled trial of mindfulness-based cognitive therapy for major depressive disorder in undergraduate students: dose-response effect, inflammatory markers and BDNF. Psychiatry Res.

[67] Kral TRA, Schuyler BS, Mumford JA, Rosenkranz MA, Lutz A, Davidson RJ (2018). Impact of short- and long-term mindfulness meditation training on amygdala reactivity to emotional stimuli. Neuroimage.

[68] Yordanova J, Kolev V, Nicolardi V (2021). Attentional and cognitive monitoring brain networks in long-term meditators depend on meditation states and expertise. Sci Rep.

[69] Schwartz K, Ganster FM, Tran US (2023). Mindfulness-based mobile apps and their impact on well-being in nonclinical populations: systematic review of randomized controlled trials. J Med Internet Res.

[70] Yosep I, Suryani S, Mediani HS, Mardhiyah A, Ibrahim K (2024). Types of digital mindfulness: improving mental health among college students - a scoping review. J Multidiscip Healthc.

[71] Macrynikola N, Mir Z, Gopal T (2024). The impact of mindfulness apps on psychological processes of change: a systematic review. Npj Ment Health Res.

[72] Ward K, Herekar A, Wang P, Lindsay KL (2023). Feasibility and acceptability of a mindfulness-based smartphone app among pregnant women with obesity. Int J Environ Res Public Health.

[73] Kubo A, Aghaee S, Kurtovich EM (2021). mHealth mindfulness intervention for women with moderate-to-moderately-severe antenatal depressive symptoms: a pilot study within an integrated health care system. Mindfulness (N Y).

[74] Porter AC, Hunter S, Noonan K, Hoffman MC (2022). A mindfulness application for reducing prenatal stress. J Midwifery Womens Health.

[75] Siegmann EM, Eichler A, Buchholz VN (2023). Effects of an app-based mindfulness intervention during pregnancy on the infant’s prenatal androgen exposure: a randomized controlled pilot trial. J Clin Med.

[76] Stentzel U, Grabe HJ, Schmidt S, Tomczyk S, van den Berg N, Beyer A (2023). Mental health-related telemedicine interventions for pregnant women and new mothers: a systematic literature review. BMC Psychiatry.

[77] Bjelica A, Cetkovic N, Trninic-Pjevic A, Mladenovic-Segedi L (2018). The phenomenon of pregnancy - a psychological view. Ginekol Pol.

[78] Khan M, Maes P Tracking diverse feelings and activities encourages self-guided holistic behavior change.

[79] Boghrati R, Sharif MA, Yousefi S, Heydarian A (2024). Emotion tracking (vs. reporting) increases the persistence of positive (vs. negative) emotions. J Exp Soc Psychol.

[80] Fredrickson BL, Boulton AJ, Firestine AM (2017). Positive emotion correlates of meditation practice: a comparison of mindfulness meditation and loving-kindness meditation. Mindfulness (N Y).

[81] Hutcherson CA, Seppala EM, Gross JJ (2008). Loving-kindness meditation increases social connectedness. Emotion.

[82] Uchino BN, Bowen K, Kent de Grey RG (2016). Loving-kindness meditation improves relationship negativity and psychological well-being: a pilot study. Psychology.

